# Staphylococcal endocarditis in a quadricuspid aortic valve following uncomplicated dengue infection: a case report

**DOI:** 10.1186/s12879-020-05315-w

**Published:** 2020-08-06

**Authors:** Nilusha Weerasooriya, Tharanga Fernando, Pasan Serasinghe, Buddhika Alahakoon, Chirath Madurapperuma, Ananda Jayanaga

**Affiliations:** National Hospital of Sri Lanka, Colombo, Sri Lanka

**Keywords:** Staphylococcal endocarditis, Quadricuspid aortic valve, Dengue fever, Linezolid, Rhabdomyolysis, Case report

## Abstract

**Background:**

Dengue fever is endemic and a leading health problem in Sri Lanka. Increased incidence of concurrent bacteremia in patients with dengue infection is a recognized complication. However, Staphylococcal endocarditis following dengue fever is uncommon. Quadricuspid aortic valve (QAV) is a rare congenital anomaly and few cases of infective endocarditis have been reported in QAV.

**Case presentation:**

A 32-year-old Sri Lankan male presented to the National Hospital of Sri Lanka with recurrence of fever and acute left hemiplegia following an uncomplicated recovery of dengue fever. He was diagnosed to have Staphylococcal infective endocarditis of quadricuspid aortic valve, with septic emboli to brain and spleen. He was managed with intravenous vancomycin initially, however, due to inadequate response, intravenous linezolid was added. He developed rhabdomyolysis with very high creatine phosphokinase leading to acute kidney injury, which settled with the cessation of linezolid. The patient succumbed to his illness despite aggressive antimicrobial therapy and maximum supportive care while being assessed for aortic valve replacement.

**Conclusions:**

This case illustrates three clinical issues that a clinician should be aware of. Firstly, the possibility of a serious secondary bacterial infection as a cause for recurrence of fever following dengue infection. Secondly, this case highlights the importance of identifying QAV as a cause for complicated infective endocarditis of increased severity. The report also denotes the value of being vigilant of linezolid induced rhabdomyolysis which had a causal relationship with the commencement of the drug and its cessation.

## Background

Dengue fever is endemic and a leading health problem in Sri Lanka. A few case series have recognized an increased incidence of concurrent bacteremia in patients with dengue infection [1, 2].

The quadricuspid aortic valve (QAV) is a rare congenital anomaly with an incidence of 0.008 to 0.043% [3]. Few cases of endocarditis in QAV have been reported [4]. We report a patient presenting with endocarditis of previously undiagnosed QAV, soon after recovering from dengue fever. The clinical course of illness was complicated by an embolic stroke and treatment-related rhabdomyolysis.

## Case presentation

A 32-year-old Sri Lankan male presented to the National Hospital of Sri Lanka with acute left hemiplegia. Two weeks before the presentation, he was treated for uncomplicated dengue fever at a local hospital. The next day of discharge from the hospital, his fever recurred. On admission to our unit, he was febrile and confused. He had a dense paralysis of the left arm and leg with a GCS of 13/15 (E-3, V-4, M-6). His pulse rate was 120 bpm with a blood pressure of 130/60 mmHg and an early diastolic murmur in the lower left sternal edge compatible with aortic regurgitation (AR) was audible.

The investigations revealed the following: white cell count-19,840/mm^3^ (neutrophils 79%) haemoglobin-14.7 g/dL, platelets-101,000/mm^3^, C reactive protein-414 mg/l. A non-contrast computed tomography of head revealed multiple hypodensities in the right cerebral hemisphere with cerebral oedema.

**Fig. 1:**
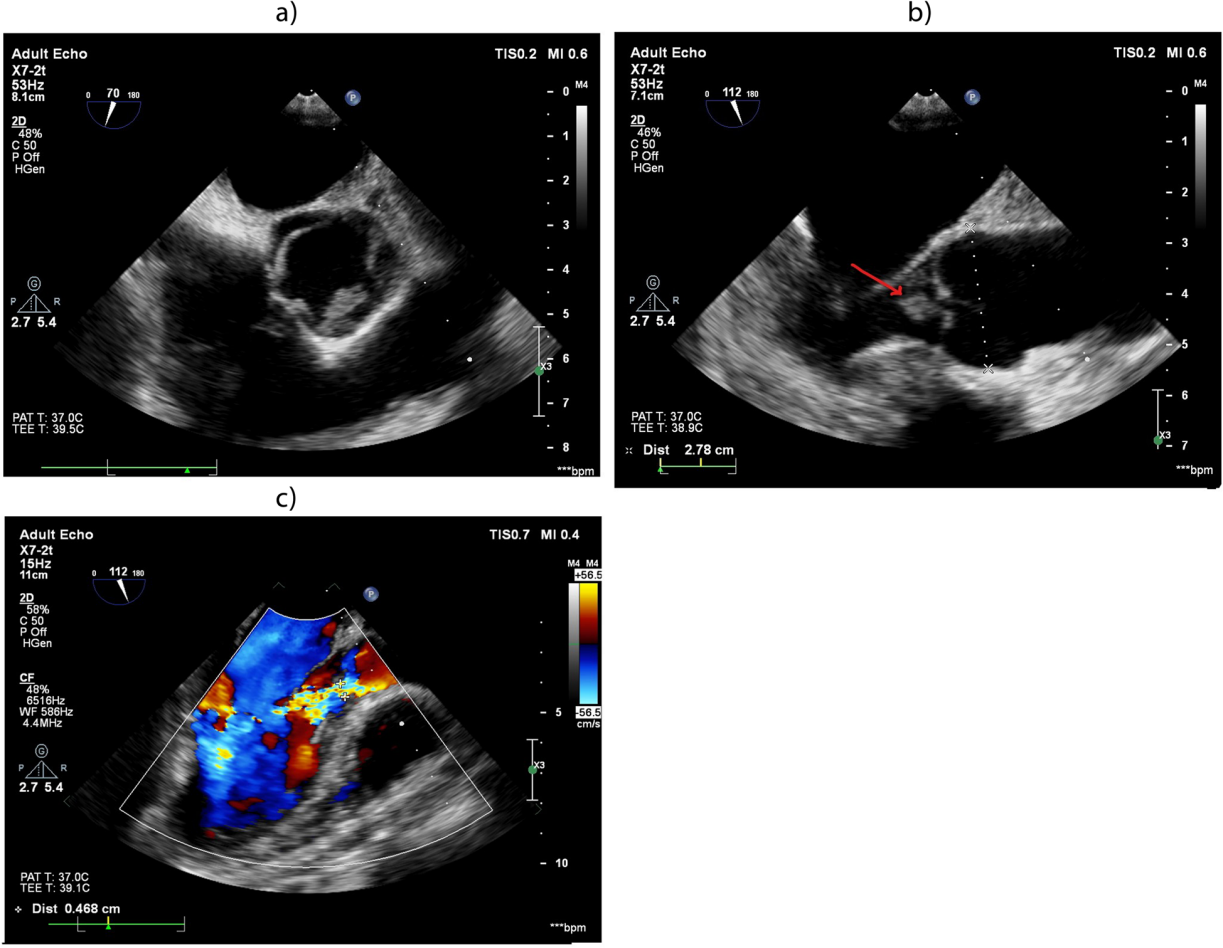
TOE, **a** Quadricuspid aortic valve, **b** Arrow, A vegetation attached to the aortic valve leaflet, **c** Doppler showing AR colour jet

The second day, intravenous vancomycin 1 g twice daily was started since his blood culture grew methicillin-resistant *Staphylococcus aureus* (MRSA), which was sensitive to vancomycin and teicoplanin. Transoesophageal echocardiogram (TOE) showed two oscillating masses (6 × 4 mm and 12 × 6 mm) separately attached to the inferior side of the aortic valve with moderate AR (Fig. 1). A diagnosis of definite infective endocarditis was established according to the modified Duke criteria. The left hemiplegia was presumed to be due to an embolic stroke and subsequently, a splenic abscess was detected ultrasonically.

Despite being on intravenous vancomycin for 4 days, his clinical status deteriorated as his consciousness further declined. The MRI brain which was done on day five revealed haemorrhagic transformation of ischaemic foci. On day six, his GCS was declining to 9/15 (E-2, V-3, M-4). He had a continuous fever and persistent sinus tachycardia of 120–130/min with the blood pressure of 110/70 mmHg maintained with 0.1 μg/kg/min intravenous noradrenaline infusion. The investigations done on the sixth day revealed no improvement: white cell count-24,000/mm^3^ (neutrophils 86%) haemoglobin-11 g/dL, platelets-94,000/mm^3^, C reactive protein-394 mg/l. Therefore, on day six, the patient required mechanical ventilation and the addition of rifampicin and intravenous linezolid 600 mg twice daily. Before commencing linezolid, on day six, his serum creatinine was 0.98 mg/dl and creatine phosphokinase (CPK) was 142 U/l. On the fourth day of linezolid therapy, his fever started to settle, however, his GCS and inflammatory markers did not improve. On the same day, his CPK rose to 10,000 U/l with a serum creatinine of 1.1 mg/dl. On the seventh day of linezolid therapy, he developed acute rhabdomyolysis with raised CPK of 104,530 U/l and acute kidney injury (AKI) (serum creatinine- 3.9 mg/dl). Furthermore, the patient’s fever recurred and he became more haemodynamically unstable, requiring escalated inotropic support. As there were reported cases of linezolid induced rhabdomyolysis, we considered linezolid as the offending agent [5]. Withdrawal of linezolid resulted in the recovery of AKI and rhabdomyolysis. Subsequently, CPK normalized. Even though he recovered from AKI, over the next 10 days, his heart failure worsened. He had persistently low GCS with recurring fever spikes complicated by ventilator-associated pneumonia and candidaemia. The patient succumbed to his illness despite aggressive antimicrobial, antifungal therapy, and maximum supportive care while being assessed for aortic valve replacement.

## Discussions and conclusions

This patient’s recovery from dengue fever was complicated with MRSA endocarditis of aortic valve with septic embolization to the brain and spleen. He was found to have QAV. We could not find any reports of increased incidence of infective endocarditis in QAV. However, QAV endocarditis has increased risk for complications such as progressive AR, decompensated heart failure, and valve perforation [4].

Hypotheses on the pathogenesis of concurrent bacteremia in patients with dengue include anti-NS1 antibody-induced endothelial cell apoptosis, which leads to endothelial dysfunction allowing bacteria to invade tissues [6]. Defective T cell activation by dengue virus-infected dendritic cells and simultaneous increase of IL-6 and IL-10, which are immunosuppressants, also contribute to the poor host response [7].

There are two reported cases of linezolid induced rhabdomyolysis [5, 8]. Rhabdomyolysis has been associated with drugs that inhibit mitochondrial function [9]. The inhibition of mitochondrial protein synthesis by linezolid may be the mechanistic cause for rhabdomyolysis [8].

This case illustrates three clinical issues that a clinician should be aware of. Firstly, the possibility of a serious secondary bacterial infection as a cause for recurrence of fever following dengue infection. Secondly, this case highlights the importance of identifying QAV as a cause for complicated infective endocarditis of increased severity. The report also denotes the value of being vigilant of linezolid induced rhabdomyolysis which had a causal relationship with the commencement of the drug and its cessation.

## Data Availability

Not applicable.

## Abbreviations

QAV: Quadricuspid aortic valve
AR: Aortic regurgitation
MRSA: Methicillin-resistant *Staphylococcus aureus*
TOE: Transoesophageal echocardiogram
CPK: Creatine phosphokinase
AKI: Acute kidney injury
